# A Real-Time Method for Time-to-Collision Estimation from Aerial Images

**DOI:** 10.3390/jimaging8030062

**Published:** 2022-03-03

**Authors:** Daniel Tøttrup, Stinus Lykke Skovgaard, Jonas le Fevre Sejersen, Rui Pimentel de Figueiredo

**Affiliations:** Department of Electrical and Computer Engineering, Aarhus University, Nordre Ringgade 1, 8000 Aarhus, Denmark; daniel.toettrup@gmail.com (D.T.); stinus2@gmail.com (S.L.S.); jonas.le.fevre@ece.au.dk (J.l.F.S.)

**Keywords:** multiple-object tracking, convolutional neural networks, time-to-collision estimation

## Abstract

Large vessels such as container ships rely on experienced pilots with extensive knowledge of the local streams and tides responsible for maneuvering the vessel to its desired location. This work proposes estimating time-to-collision (TTC) between moving objects (i.e., vessels) using real-time video data captured from aerial drones in dynamic maritime environments. Our deep-learning-based methods utilize features optimized with realistic virtually generated data for reliable and robust object detection, segmentation, and tracking. Furthermore, we use rotated bounding box representations, obtained from fine semantic segmentation of objects, for enhanced TTC estimation accuracy. We intuitively present collision estimates as collision arrows that gradually change color to red to indicate an imminent collision. Experiments conducted in a realistic dockyard virtual environment show that our approaches precisely, robustly, and efficiently predict TTC between dynamic objects seen from a top-view, with a mean error and a standard deviation of **0.358** and **0.114** s, respectively, in a worst-case scenario.

## 1. Introduction

Maritime environments are increasingly populated with vessels, which must have a trained nautical pilot on board responsible for safely maneuvering the ship to its destination [1]. Nevertheless, safety inside dockyards is hard to ensure and accidents due to human errors can be costly or deadly. An assisting tool working autonomously to support the maritime pilot by predicting possible collisions would lower the risk of navigating the vessel [2,3].

With a companion unmanned aerial vehicle (UAV), one may prevent potential collisions and reduce vulnerabilities in waterway environments. By using artificial intelligence (AI), autonomous systems must be capable of estimating when hazardous scenarios may occur and provide helpful information to vessel and shipyard personnel for improved decision-making to prevent physical and human losses. By using advanced deep learning techniques to produce high-quality, real-time collision predictions, it is possible to assist harbor personnel in ensuring a safer harbor environment for ships and staff.

The main goal of this work is to use UAVs and state-of-the-art machine vision and learning algorithms to improve navigation safety and effectiveness in dockyard environments. We propose algorithms for an autonomous UAV and a monocular camera directed downwards to predict time-to-collision (TTC) between ships in water environments. For a UAV to correctly operate within a new environment, large amounts of image data from the environment are needed for training. These training data are needed to help the AI gain contextual knowledge of the environment so it can make correct predictions. We use advanced simulation tools that simulate various maritime environments to collect large amounts of data on demand. Furthermore, this environment can be used to validate and test the system to obtain a qualitative understanding of our algorithms performance.

**Figure 1:**
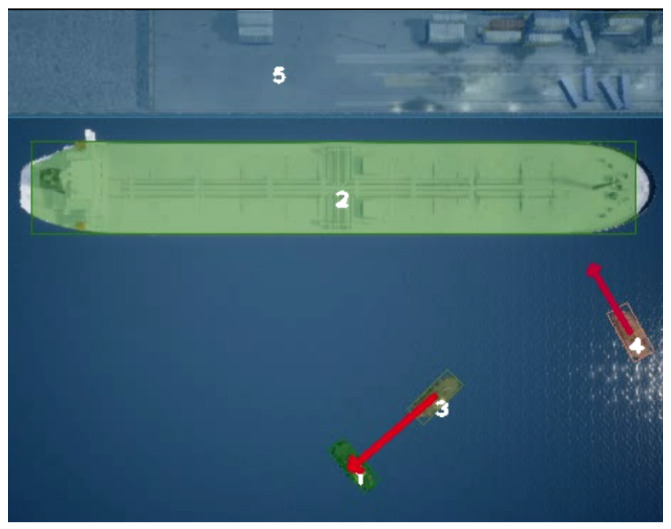
Image of four vessels in collision route gathered by an autonomous UAV, and TTC estimated using our real-time system.

To ensure safe navigation in water environments, the main contribution of the present work, concerning our previous work [3], resides on producing reliable TTC estimations from top-view video streams (see Figure 1). More specifically, we extend a data-driven DNN semantic segmentation approach for precise object localization utilizing video streams acquired from aerial top-views, and a robust deep-learning-based tracking method, which ensures reliable data association of detections across frames of video streams, using refined rotated bounding boxes for accurate multiple-object tracking. The main contribution of this work is a novel accurate method for TTC estimation between all maritime vehicles (i.e., vessels) within harbor environments that utilizes semantic segmentation and aligned bounding box representations, and a visualization interface to present TTC in an easy-to-understand manner.

The remainder of this paper is outlined as follows. First, we overview the related work in object detection, segmentation, and tracking approaches, then we present the developed approaches for estimating TTC of objects in shipyard contexts using top-view images obtained from a drone.

## 2. Related Work

In the remainder of this section, we overview the state-of-the-art in machine learning and vision approaches to solve the TTC estimation problem, with an emphasis on object detection, segmentation, tracking, collision avoidance, and TTC estimation.

### 2.1. Object Detection and Semantic Segmentation

Object detection aims at determining where (localization) and what (classification) objects reside in images [4]. Classical approaches attempted for object detection problem can be decomposed in the following main steps: region selection, feature extraction, and classification. However, due to the work of Krizhevsky et al. [5], AlexNet, an end-to-end deep convolutional neural network (DCNN) approach for visual classification, achieved top accuracy in the large-scale visual recognition challenge (ILSVRC) [6]. In [7], the deep-learning based object detection method named Fast R-CNN that speeds up object detection introduced the idea of using multi-scale pooling of images [8] and a single passage over the entire input image, succeeded by a region of interest (ROI)-pooling layer that divides the ROI into a fixed size, allowing to feed input images of arbitrary size. The computational bottleneck of Fast R-CNN is the region proposal algorithm, which, like the original R-CNN, utilizes selective search to generate region proposals. In Faster R-CNN [9], the authors proposed further extensions to the previous approach. Namely, the RPN (region proposal network) that jointly estimates object bounds and scores at each image location, reducing the region proposals’ computation times. The state-of-the-art work of [10] proposed a faster and more precise object detection neural network named YoloV4 than any other available real-time object detectors at the time.

While object detection provides each instance and location of a class, semantic segmentation provides a pixel-level classification of all pixels in an image. On the other hand, instance segmentation includes identification and spatial localization of objects using bound boxes, and within each bounding box, the binary semantic segment of each pixel. In [11], a further extension of the previously mentioned Faster R-CNN called Mask R-CNN is introduced. Mask R-CNN adopts the same architecture and structure from Faster R-CNN, but has significant improvements. The authors extend Faster R-CNN by adding a branch for binary object mask prediction, in parallel with the existing branch for bounding box estimation. In [12], a network called fast segmentation convolutional neural network was introduced, which is suitable for embedded devices with low processing and memory specifications. The network comprises four modules: Learning to Down-sample, Global Feature Extractor, Feature Fusion, and Classifier. The network performs above real-time (123.5) fps for high-resolution images (1024 × 2048 px) and implements skip-connections popular in offline DCNNs with the shallow *learning to downsample* module. The Fast SCNN network exhibits run-time improvements with a minor loss in accuracy when compared with the previous semantic segmentation approaches. These data-hungry methods are typically trained with simulation data and adapted to reality using domain randomization and adaptation techniques [13,14].

### 2.2. Multiple-Object Tracking

Multiple-object tracking (MOT) tackles the association of object detection across video frames to estimate and maintain object trajectories and identities over time simultaneously. In this work, we employ the widely used a simple, online, and real-time tracking (SORT) algorithm [15], which performs recursive state estimation via Kalman filtering in image coordinates. Object detections association (i.e., data association) is achieved via the Hungarian method with an association metric that assesses bounding box overlap. DeepSORT [15] combines the former method with robust appearance-based features extracted with deep-learning methods, allowing tracking objects through long periods of occlusion, and hence reducing the amount of identity switches. Much of the computational complexity was transferred to an offline pre-training phase in which a deep association metric is learned on re-identification datasets to minimize inference time. The work of [16] introduces an approach named Track R-CNN that simultaneously solves detection, tracking, and segmentation, demonstrating that bounding-box tracking performance improvements are attained when using fine classification (i.e., at the pixel level). Track R-CNN, a method based on Mask R-CNN, extends the later by incorporating the time dimension through 3D convolutions to associate object identities over time. While the previous online greedy approaches employ recursive inference techniques, for sequentially arriving images, offline techniques optimize trajectories and identities for image batches, in a global manner. These approaches use formulated multi-frame and multi-target data association as a graph optimization technique [17,18].

### 2.3. Collision Avoidance

TTC was first introduced in [19] and deals with the problem of estimating the time duration before two or more objects collide, given some certain initial conditions. In the work of [20], the authors propose a method to calculate TTC between two vehicles to improve vehicle safety. In [21], the authors introduced a system that, with the use of a unmanned aerial surveillance system (UASS), can autonomously recognize objects in the path of a traversing vessel at sea, resulting in a collision. The UASS sends information to the vessels. Proper collision avoidance actions are made, and collision avoidance maneuvers are taken considering the convention on the international regulations for preventing collisions at sea (COLREGs) rules. The UASS detects objects using machine learning techniques. The simulation results show that such a system is feasible and promising in assisting the vessels to avoid obstacles by using a small drone scout. However, no details of the detection techniques deployed on the system were provided, and many simplifying assumptions were made. In [22], the authors proposed a method for forecasting the TTC using only a single monocular camera and a convolutional neural network (CNN) to process the image data. A camera mounted on top of a suitcase-shaped robot and a CNN are used to predict when the robot will collide with objects in the viewpoint of the mounted camera. Furthermore, they produced a large dataset to train their network, with ground truth annotations obtained using LIDAR data. Their results show that using a mounted camera and predicting the TTC in a first-person view and using CNNs to make predictions is promising and a relevant direction to forecast time to near-collision. Likewise, ships seen from a top (aerial) view can be enclosed by rectangular bounding boxes in the image plane.

## 3. Methodologies

**Figure 2:**
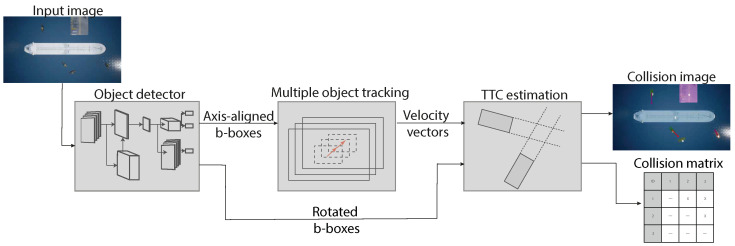
The individual modules that compose our system. The object detection module takes an image as input and outputs bounding boxes and segmentation masks.The second module (object tracker) receives output from object detector and performs tracking of these over time. The third module utilizes output estimated bounding boxes of multiple-object trackers to estimate TTC between all tracked objects.

This section describes in detail our framework for TTC estimation between vehicles seen from aerial images. Moreover, we overview our approach for generating a training and validation dataset. Figure 2 depicts the proposed architecture, showcasing how each module interacts with the others.

### 3.1. Vessel Detection from Drone Aerial Images

The end goal of the proposed system is to be able to reliably estimate when ships will collide with other ships, using a single UAV with a camera attached to the underside pointing downwards. The system uses bounding boxes to represent the objects’ states and to estimate whether and when these will collide.

**Figure 3:**
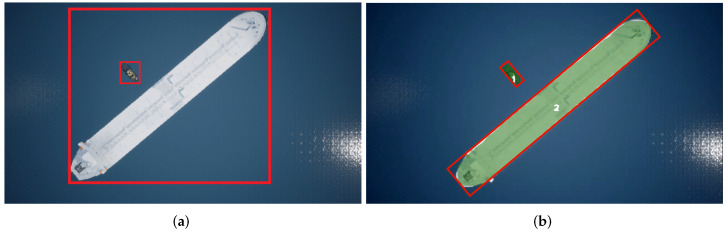
(**a**) Axis-aligned bounding boxes where the bounding boxes of two ships intersect. (**b**) A rotated bounding box computed from pixel-level segmentation (and the corresponding segmentation masks superimposed on top of both ships), that allows representing vessel’s spatial location more accurately and avoid bounding box intersections (figure reproduced from [3]).

In this work, the widely used YoloV4 [10] and Faster R-CNN [9] were considered as candidates for object detection. However like other standard object detectors, these create axis-aligned bounding boxes around the objects of interest. As almost all ships have an elongated shape, an axis-aligned bounding box can negatively impact the ability to estimate a TTC. This is caused by the fact that when a ship is located diagonally in the image frame, the output from an object detector will be a square bounding box, which is not tightly fit to the ship (see Figure 3). To overcome this issue, instance-based semantic segmentation was preferred since it allows for more accurate representations of the detected objects in an image, as each pixel in the image belongs to a class. However, since the TTC algorithm uses an encompassing box around the vessel of interest, a new fitted bounding box needs to be produced based on the segmentation. To perform this, an algorithm called rotating calipers [23] is used, which finds the smallest possible rectangle that can fit a given convex hull, which in our case is the segmentation result (see Figure 3). Details about this algorithm will be discussed in Section 3.2.

### 3.2. Multiple-Object Tracking from Top-Views

For tracking, we rely on the recursive Bayesian estimation-based algorithm named Deep SORT [24]. Deep SORT relies on Kalman filtering and a frame-by-frame data association approach. As illustrated in Figure 2, the object detector bounding boxes and the associated segmentation masks at each frame outputted from the detector are fed to the tracker. Low confidence score detections are filtered out using a confidence score threshold to reduce the amount of false positives. Non-maximum suppression is a common approach to avoid multiple bounding boxes for the same object. Each detection, starting with the highest confidence score one, is compared with all the others through the intersection over union (IoU). If the IoU is above a given user-specified threshold, the bounding box with the lowest score is disregarded. Our method relies on aerial 2D image views, thus we assume that objects never overlap. Hence, one should set the IoU threshold overlap to a low value (e.g., 0.1 ). The rotated bounding boxes are used for non-maximum suppression. They provide a more accurate representation of the object location, thus allowing lower IoU thresholds, without erroneously suppressing correct bounding boxes. After filtering the detections, the remaining bounding boxes are fed to the Deep SORT algorithm as (u,v,γ,h) , where (u,v) represents the center coordinate of the bounding box, γ the aspect ratio, and *h* the height.

The tracking framework implemented by Deep SORT is a constant velocity Kalman filter, defined as an eight-dimensional state vector $\left(u,v,\gamma ,h,\dot{x},\dot{y},\dot{\gamma },\dot{h}\right)$ for recursive object state estimation. The data association problem between predictions (i.e., Kalman states) and observed states (i.e., detections), both motion and appearance information are used using the Hungarian method. The motion information is filtered considering the Mahalanobis distance [25] between the predicted Kalman states and the measured states. The Mahalanobis distance is a good association metric when motion uncertainty is well modeled; however, fast camera motion or temporary occlusions may introduce unpredictable and fast changes in the image location of objects from frame to frame. In these cases, the Mahalanobis distance metric may become unsuitable for accurate tracking. Therefore, we also consider an additional metric based on the objects’ visual appearances. For each bounding box, an appearance descriptor is obtained via pre-trained convolutional neural network (CNN) comprising two convolution layers (one max pooling and a six-residual block layer) and a fully connected layer that outputs a 128-dimension feature vector. The CNN assigned to generate appearance descriptors is trained on 40 instances of each class. The appearance metric is given by the smallest cosine distance between tracked and measured bounding boxes (see [3]).

### 3.3. Time-to-Collision Estimation

**Figure 4:**
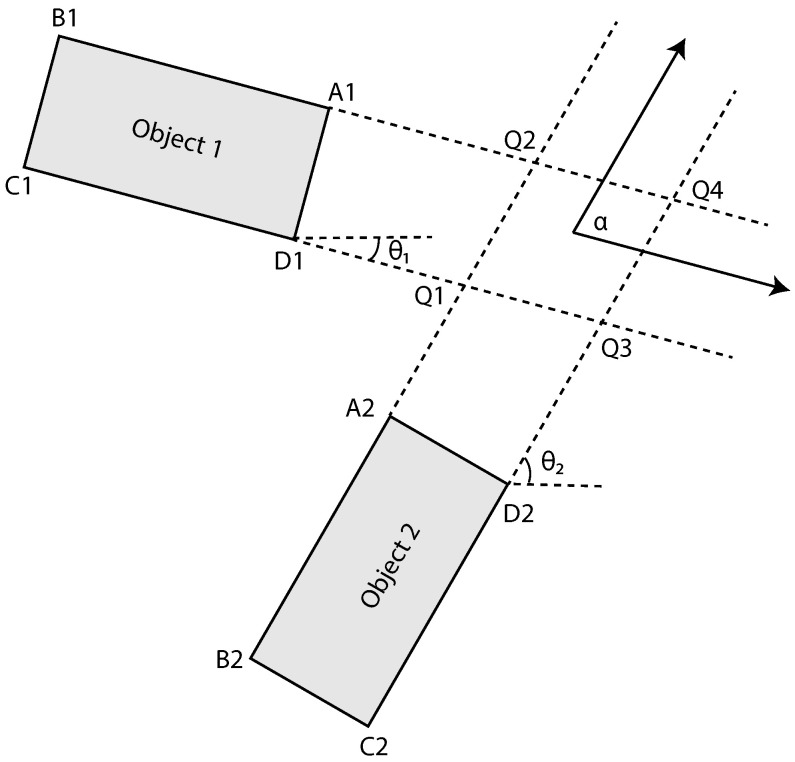
Illustration of intersection point between two moving objects. A1, B1, C1, D1, A2, B2, C2, and D2 represent corner point coordinates of two objects. Q1, Q2, Q3, and Q4 represent intersection coordinates. α represents collision angle between two objects.

The method from [20] was chosen to compute the TTC between dynamic objects. In this method, the authors proposed an improved method to calculate the TTC compared to that of the prior method proposed in [26]. The improved method builds on the knowledge that, when two objects collide, a corner of one of the objects will be the first area that comes into contact. Unless the collision is perfectly perpendicular, then the two corners will come into contact at the same time. Therefore, by calculating the intersection points aligned with the corners of the two objects, the first point of iteration can be calculated. The intersection point is calculated according to the following:

(1) $$x_{+}=\frac{\left(y_{2}-y_{1}\right)-\left(x_{2}\cdot tan\theta_{2}-x_{1}\cdot tan\theta_{1}\right)}{tan\theta_{1}-tan\theta_{2}}$$

(2) $$y_{+}=\frac{\left(x_{2}-x_{1}\right)-\left(y_{2}\cdot cot\theta_{2}-y_{1}\cdot cot\theta_{1}\right)}{cot\theta_{1}-cot\theta_{2}}$$

where $x_{+}$ and $y_{+}$ represent the intersection coordinates, $x_{1}$ , $y_{1}$ , $x_{2}$ and $y_{2}$ the corner coordinates, and $\theta_{1}$ and $\theta_{2}$ the direction of the objects 1 and 2, respectively (see Figure 4). The four intersection points are computed for the corner points of the two objects. This is illustrated in Figure 4 where the intersection between points Q1 to Q4 and α represent the collision angle between the two objects, calculated as $\alpha =\theta_{1}-\theta_{2}$ . This is illustrated in Figure 4, where the intersection points are Q1 to Q4. These four intersection points result in 32 possible collision scenarios, as the four corners of an object can impact on any four sides of the other object. However, out of these 32 situations, only 10 are possible. TTC estimation requires computing the time for all corner points of both objects to reach the four intersection points, resulting in 16 TTC values. The 10 possible situations are then divided into two initial configurations, if the $\alpha <90^{\circ }$ and $\alpha >90^{\circ }$ . In [20], they presented two tables corresponding to the two initial configurations with a total of 10 collision conditions. The 16 time values are compared with the 10 collision conditions to find a match and estimate the TTC between the objects. The shortest time for a corner point of the moving object to reach an intersection on the stationary object is then estimated as the TTC between the two objects, using the output of a multiple-object tracker.

The expected time-to-intersection between the two objects is computed according to:

(3) $$\begin{array}{c} {\mathrm{TTX}}_{1}=\frac{|{\vec{r}}_{+}-{\vec{r}}_{1}|}{|{\vec{v}}_{1}|}\mathrm{sign}\left(\left({\vec{r}}_{+}-{\vec{r}}_{1}\right)\cdot {\vec{v}}_{1}\right) \end{array}$$

(4) $$\begin{array}{c} {\mathrm{TTX}}_{2}=\frac{|{\vec{r}}_{+}-{\vec{r}}_{2}|}{|{\vec{v}}_{2}|}\mathrm{sign}\left(\left({\vec{r}}_{+}-{\vec{r}}_{2}\right)\cdot {\vec{v}}_{2}\right) \end{array}$$

where ${\vec{v}}_{1}$ and ${\vec{v}}_{2}$ represent the velocities of the objects, ${\vec{r}}_{n}$ represents the coordinate vector $\left(x_{n},y_{n}\right)$ , and sign the sign function.

If the two objects obtain to their intersection point at the same time, i.e., TTX1=TTX2 , then there is an expected TTC. We utilize the simplifying squared objects assumptions defined in [20], to provide more accurate TTC estimates.

## 4. Results

In this section, we assess the performance of our MOT and TTC estimation pipeline using a dataset obtained using a realistic simulation shipyard scenario that was modeled using AirSim [27]. The UAV is equipped with a monocular camera with no lens distortion.

### 4.1. Benchmarking Metrics

Next, we introduce utilized metrics to assess the performance of our solutions.

#### 4.1.1. Multiple-Object Tracing Accuracy (MOTA)

The multiple-object tracking accuracy (MOTA) [28] is a commonly used metric to assess multiple-object tracking performance, by combining three sources of errors: the number of false positives, false negatives, and ID switches, according to:

(5) $$MOTA=1-\frac{\sum_{t}\left(FN_{t}+FP_{t}+IDSW_{t}\right)}{\sum_{t}GT_{t}}$$

where FN represents the false negatives, FP the false positives, IDSW the number of ID switches, GT the ground truth, and *t* the frame index. MOTA results can range from ( −∞ , 100% ), and negative values may only occur if the number of tracking mistakes exceed the number of objects in the scene [29].

#### 4.1.2. Multiple-Object Tracking Precision (MOTP)

Multiple-Object Tracking Precision (MOTP) [28] is used as a precision metric for the tracker evaluation. This is performed by computing the average dissimilarity between true positives and the corresponding ground truth, according to:

(6) $$MOTP=\frac{\sum_{t,i}d_{t,i}}{\sum_{t}c_{t}}$$

where $c_{t}$ is the number of matches in frame t and $d_{t,i}$ is the overlap between target *i* and its assigned ground truth object. In other words, multiple-object tracking precision (MOTP) gives an average overlap between all correctly matched bounding boxes and their respective ground truth objects [29].

#### 4.1.3. Mean Squared Error (MSE)

The mean squared error (MSE) is a measure of the average squared errors obtained. The measure is the mean error between the estimated values and the actual values over time. This metric assesses how far the predicted measurements are from the ground truth values on average:

(7) $$MSE=\frac{1}{n}\sum_{i=1}^{n}{\left(Y_{i}-{\hat{Y}}_{i}\right)}^{2}$$

where *n* represents the number of predictions, *i* is the given time instance, *Y* represents the predicted values, and $\hat{Y}$ represents the ground truth values. This measurement will be used to evaluate the TTC estimation as a metric to evaluate the prediction accuracy.

### 4.2. MOT Performance Evaluation

First, we assess the performance of the multi-object tracking approach using four different scenarios, with increasing levels of difficulty. The first two scenarios involve the UAV hovering at a stationary point (static viewpoint), where no occlusions disturb the camera’s view. The third consists of an object continuously occluding the view of the camera. The fourth and final scenario involves the UAV flying around in the simulated environment. The objective of the first two scenarios is to evaluate the tracker’s performance in ideal circumstances when no collision and changes of the viewpoint are applied. The objective of scenarios three and four are to evaluate the tracker under challenging scenarios. To assess the tracker’s performance, we use the standard multiple-object tracking evaluation metrics [29], MOTA and MOTP, to obtain the number of true positives, false positives, false negatives, and ID switches.

Table 1 shows that the tracker performs robustly when the camera viewpoint is fixed with a score above 90 on both MOTA and MOTP. The tracker has the hardest time tracking objects in scenario three, where occlusions are applied. This is due to the tracker wrongfully detecting the flying occlusion object as class 1, resulting in increasing false positives. The tracker performs well on scenario four and shows that the tracking performance is not disturbed by the movement of the UAV, demonstrating the robustness of the tracker to cases where the image plane is not parallel to the water surface.

We note, however, that our framework could benefit from more recent globally optimal tracking approaches based on network flow formulations and minimum-cost flow solvers [18], instead of data association performed on a greedy frame-by-frame basis, to reduce ID switches and improve trajectory smoothness.

### 4.3. TTC Performance Evaluation

**Figure 5:**
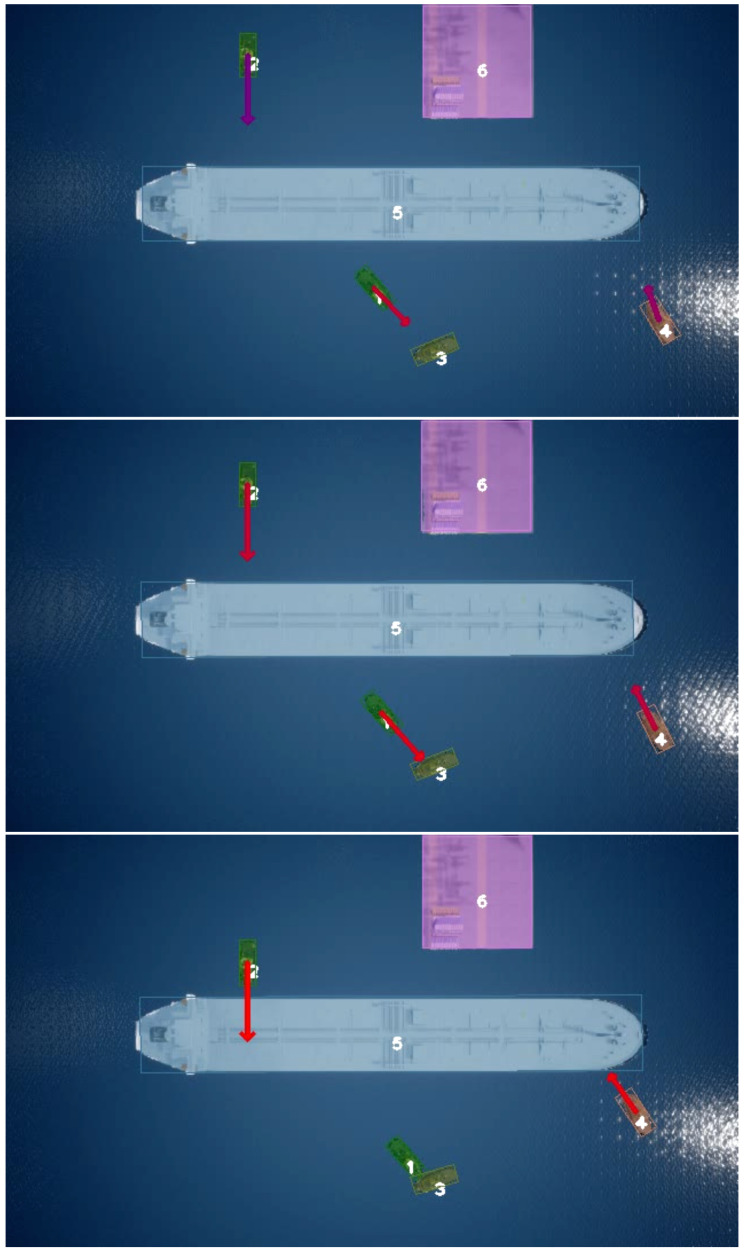
Sequential image samples of output of our TTC estimation approach. Each vessel is colored with a translucent color that corresponds to its segmentation mask.

To validate the performance of the proposed TTC approach, we first investigate how the angle between two objects on a collision course influences the TTC estimation precision. We calculate the mean error and standard deviation of each test TTC to evaluate the accuracy. An example sequence of frames taken at different time intervals of the output of our TTC methodology can be seen in Figure 5.

**Figure 6:**
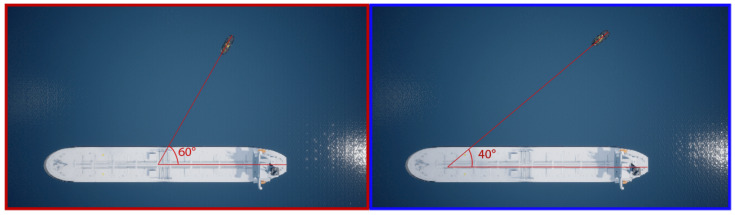
Examples of collision angle test between two vessels on a collision course. (**Left**) Highlights in red represent the test with vessels experiencing a collision angle of 60 ${}^{\circ }$ . (**Right**) Highlights in blue represent the two vessels experiencing a collision angle of 40 ${}^{\circ }$ .

Figure 6 depicts an example of a simulated environment, where the collision between two objects types—class 1 (large vessel) and class 2 (tugboat)—is validated. The large vessel is stationary (0 velocity), and the tugboat is moving.

We showcase how the performance of the TTC estimation handles multiple collisions in an all vs. all scenario. This experiment, like the others, is constructed in a simulated environment. The scenario involves seven objects in the scene: one of the objects is at a fixed point, and the others are moving at a constant speed in a fixed direction. In each frame, the predicted TTC between all objects is compared to the ground truth TTC. The mean error between the estimated and ground truth TTC and the standard deviation is used for performance assessment. Table 2 shows an example scenario where our framework estimates that four collisions between the seven dynamic objects in the scene will occur.

**Figure 7:**
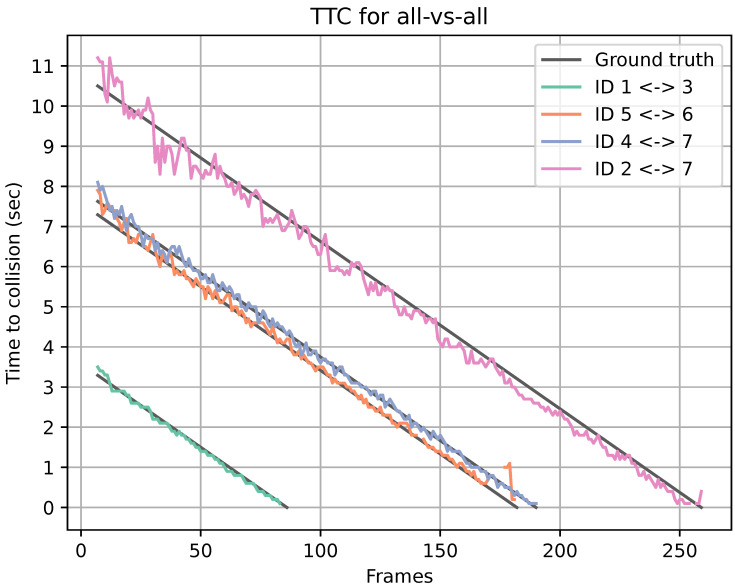
Predicted TTC for all four collisions from scenario 1, and corresponding ground truth TTC.

Figure 7 shows the predicted TTC for all four collisions and the corresponding ground truth TTC from scenario one. The missed estimations are represented as noncontinuous data, and the line has a gap. The line chart shows a good estimation of the TTC as the estimations and ground truths follow each other. The missed estimations shown in Table 3 are all located at the end of each prediction. This could result from the bounding boxes of the objects having already collided. The average TTC mean error and standard deviation of all collisions within the scene are shown in Table 2.

## 5. Conclusions

In this work, we introduced a framework for multiple-object detection and tracking and time-to-collision estimation of maritime vehicles, from top-view video streams using appearance features extracted with deep learning techniques. Our experiments conducted in a virtual realistic environment validated the usability of our system.

Our method uses rotated bounding box representations for enhanced TTC estimation accuracy. Collision estimations are presented in a real-time visual manner, as collision arrows that gradually change their color to red to indicate increasingly potential collisions. Experiments in a dockyard virtual environment show that our approach can accurately, robustly, and quickly predict TTC between dynamic objects seen from a top-view, with mean error and standard deviation of 0.358 and 0.114 s, respectively, in a worst case scenario.

We note that the proposed system may also be used to assist large vessels when sailing through critical and narrow passages, and is not constrained to be used in maritime environments. Furthermore, although the proposed system was developed to operate in maritime contexts, it may be easily adapted to other domains, if domain-specific training data is provided, namely traffic monitoring.

## Figures and Tables

**Table 1:** Combined results of our tracking methodology evaluated in four scenarios (results reproduced from our experiments published in our previous work [3]).

Scenario No.	MOTA	MOTP	TP	FN	FP	IDSW
1	98.55	93.82	5515	55	22	4
2	97.52	90.29	6058	69	62	21
3	74.65	88.22	4388	302	536	351
4	89.00	86.59	3141	199	111	65
Combined	90.89	87.98	19102	625	497	441

**Table 2:** Average TTC mean error and standard deviation over an entire sequence of all collisions for an example scene where "X" represents no crossing paths.

IDS	1	2	3	4	5	6	7
1	-	X	0.045 ± 0.049	X	X	X	X
2	-	-	X	X	X	X	0.237 ± 0.170
3	-	-	-	X	X	X	X
4	-	-	-	-	X	X	0.075 ± 0.075
5	-	-	-	-	-	0.113 ± 0.177	X
6	-	-	-	-	-	-	X
7	-	-	-	-	-	-	-

**Table 3:** Number of missed TTC estimations for each collision between two objects together with total number of frames before a collision happens in a scenario.

Collisions	No. Missed TTC Estimations	Total Frames
ID 1 ↔ ID 3	3	81
ID 2 ↔ ID 7	2	254
ID 4 ↔ ID 7	0	185
ID 5 ↔ ID 6	9	177

## Data Availability

Not applicable.
