# Correction: Tøttrup et al. A Real-Time Method for Time-to-Collision Estimation from Aerial Images. *J. Imaging* 2022, *8*, 62

**DOI:** 10.3390/jimaging8090241

**Published:** 2022-09-06

**Authors:** Daniel Tøttrup, Stinus Lykke Skovgaard, Jonas le Fevre Sejersen, Rui Pimentel de Figueiredo

**Affiliations:** Department of Electrical and Computer Engineering, Aarhus University, Nordre Ringgade 1, 8000 Aarhus, Denmark

The authors wish to make the following corrections to the article [1]. Parts of the Abstract, Introduction, Related Work, Methodology, Results and Conclusions sections were rephrased to reduce the overlap with our previous work [2]. In particular, we changed the following:

1. In the Introduction, we better outlined our contributions with respect to our previous work, emphasizing that our main contribution concerns TTC estimation from aerial images.
2. In the methodology Section 3.2, we shortened the last paragraph, removed Equation (1) and referred to our previous work [2], focusing on our novel contributions to TTC estimation from aerial images.
3. Figure 3 was reproduced from [2] to facilitate the explanation of the novel methodologies. We added a sentence (figure reproduced from [2]) to the end of the caption.
4. The results in Table 1 were reproduced from experiments published in our previous work [2] (see Table 10).

The authors state that the scientific conclusions are unaffected. The original publication has also been updated.
